# The relative impact of barriers to care among military health services personnel: exploring differences using context specific scenarios

**DOI:** 10.1186/s12913-022-07850-5

**Published:** 2022-05-06

**Authors:** Jennifer Born, Christine Frank

**Affiliations:** Director General Military Personnel Research and Analysis, Department of National Defence, National Defence Headquarters, 101 Colonel By Drive, Ottawa, ON K1A 0K2 Canada

**Keywords:** Access to care, Providers, Military, Mental health, Physical health

## Abstract

**Background:**

Military health care providers often under access both physical and mental health care, yet research has predominantly focused on barriers to mental health care. This study explored a comprehensive set of barriers using hypothetical scenarios to quantify barrier impact on access to both mental and physical health care.

**Methods:**

Canadian military health services personnel (*N* = 1033) completed one of two electronic surveys (assessing either physical health or other mental health barriers) that captured participant’s demographics, health, endorsement of barriers, intent to seek care, and whether the respondent would access care in different health scenarios (pneumonia, back injury, depression and post-traumatic stress disorder). Logistic regression was used to calculate odds of not accessing care (versus accessing care) for each of the four health scenarios.

**Results:**

All barrier factors independently predicted increased odds of not accessing care for all four scenarios. When entered into an adjusted model none of the barrier factors significantly predicted accessing care in the physical health scenarios. Staffing and workload resources and Treatment preferences (e.g., self-treat) were significant predictors of accessing care in the mental health scenarios. Weak general intentions to access care was the strongest predictors of not accessing care across all four scenarios.

**Conclusions:**

The impact of barriers on hypothetical care-seeking behaviour differs depending on the context for which one is accessing care, with access to resources and preference to self-treat driving mental health care seeking. Intent appears to be the most impactful predictor of accessing care potentially mediating the effect of other barrier types on care seeking.

**Supplementary Information:**

The online version contains supplementary material available at 10.1186/s12913-022-07850-5.

## Background

Similar to their civilian counterparts, military health care providers (HCPs) work under physically and mentally demanding conditions [1] and are at increased risk of experiencing mental and physical health issues [2, 3]. Additionally, military HCPs are exposed to unique stressors related to the military environment in which they operate [4, 5]. In the Canadian Armed Forces (CAF), members access primary care through the military system, and only access the publically funded provincial health care systems after hours or for services not provided in-clinic (e.g., MRIs). To consult a military health care provider, appointments can be made with the individual’s Care Delivery Unit or members can drop-in to a daily sick parade (similar to a walk-in clinic). Thus, unlike other CAF personnel, CAF HCPs are often required to access care at their place of work, to sit in the same waiting room as their patients, receive care from, and have their health information shared with, colleagues, and may potentially even be required to share personal health details with members of their chain of command. In addition, if a health care provider accesses care, they potentially put a disproportionate strain on the medical system. For example, if there are five patients waiting to be seen and two physicians providing care; if one physician needs to access care, now there are six patients waiting and only one physician. Care providers must balance their need for care against providing care for others [6].

Evidence suggests military personnel [7] and civilian health care providers [8] do not access care when they should. Not accessing care or delaying treatment can lead to worse health outcomes for the provider, but can also put their patients at risk [9, 10]. This is especially concerning for military HCPs who are directly responsible for the health of military personnel, and indirectly responsible for the operational effectiveness of the entire organisation [11].

Often, failure to seek care is due to a denial of the need for treatment as well as, or in addition to, perceived barriers to care [12, 13]. Thus, reducing or removing barriers should positively impact accessing care. However, it is essential to first identify and quantify existing barriers. There are many scales used to measure barriers to care among of civilian providers (reviewed by Kay and colleagues [14]) and a more limited number used to measure barriers to care among military members (e.g., Hoge and colleagues [15], Britt and colleagues [16], and Sudom and colleagues [17]). There are several issues with these scales that need to be addressed: (1) there is little consistency between the scales; (2) they tend to focus on stigma-related barriers especially toward mental health service use within military populations; and perhaps most notably (3) the development of items lacks an underlying theoretical base [18]. Scales developed without theory, risk omitting key psychological constructs related to behaviour. Scales guided by theory have the added benefit of an empirical foundation to link behavioural change techniques to address barriers based on an understanding of their psychological underpinnings [19]. Developing interventions is difficult, but choosing evidence based methods are preferred over the all too common ISLAGIATT principle, “It seemed like a good idea at the time” [20]. Interventions developed to improve access to care that systematically address the known psychological, social or environmental barrier factors are poised to have a higher probability of success [21].

With this in mind, our preliminary research on barriers to care, used focus group discussions with our target population to develop and validate a comprehensive measure of barriers based on domains covered in Michie and colleagues' theoretical domain framework (TDF, [22]). In an attempt to create a comprehensive framework Michie and colleagues first identified 128 constructs that may influence behaviour at the motivational (i.e., theories that explain behaviour change in those who have no established intent to change), action (i.e., theories that explain the behaviour of those motivated to change), and organizational (i.e., theories to explain change at the social and organizational level) theory groups. Discussion among subject matter experts (i.e., health psychologists) resulted in the selection of a smaller set of 12 constructs that were determined to be particularly relevant to changing the behavior of healthcare professionals (i.e., knowledge; skills; social/professional role and identity; beliefs about capabilities; beliefs about consequences; motivation and goals; memory, attention and decision processes; environmental context and resources; social influences; emotion; behavioral regulation; and nature of the behaviors; see Michie et al. [22] for an overview of the original 12 domains). Later, these domains and further nested onto what Michie and colleagues (2011) classified as the COM-B system [23] in which Capability (e.g., knowledge and skills); Opportunity (factors outside the individual) and Motivation (habitual processes, emotional responding, and analytical decision-making) influence behaviour.

Validation work on the theoretically-grounded barrier scale identified eight barrier factors and that were then mapped onto the COM-B system [23]: Capability (1) Knowledge and ability to access care; Opportunity (2) Staffing and workload resources, (3) Organizational & social support; and Motivation (4) CAF HCP identity, (5) Discomfort accessing care at work, (6) Conflicts with career goals, (7) Treatment preferences (e.g., self-treat), and (8) Concerns about privacy (see Frank & Born, [24] for details). Many of the barriers types identified in our earlier research have not been examined in past barrier research. For example, the barrier scale developed by Hoge and colleagues [15] did not assess barriers related to a preference to self-treat, concerns about privacy, or a lack of social support. The barriers measured by Britt and colleagues [16] did not include barriers related to knowledge of how to access care, resources such as the time to access care, or having to access care where one works. Last, the study by Sudom and colleagues [17] did not access items related to privacy, or preferences to self-treat.

Aside from a recent publication by Britt and colleagues [16], most research on barriers to care in military populations have focused exclusively on barriers to mental health care. Additionally, numerous studies have quantified the prevalence of different types of barriers (i.e., whether that barrier exists) among both providers [14] and military personnel [25], but there is limited information, particularly for physical health, about the relative impact of those barriers (i.e., whether the barrier inhibits care seeking), and how best to mitigate the impact of those barriers in order to improve care. There is evidence to suggest that stigma is experienced in military populations [25] and that additional systemic barriers are experienced by providers [14], but few studies compare the relative contribution of different types of barriers. In one such study, Sudom et al., [17] reported that though stigma was associated with having a need for mental health care in military members, it had no association with care seeking-propensity; whereas structural barriers and negative attitudes toward care were more likely to affect mental health care seeking. This study differs from the current one in two ways: (1) Sudom and colleagues [17] used a small set of barrier items that were not theoretically grounded and (2) they only examined the relative impact of barriers on mental health care access and did not examine the impact on access to care for physical health.

Another consideration when exploring access to care is the role of intent. A review by Sheeran [26] estimated that intention explained over a quarter of the variance in behaviour across a range of contexts. A meta-analysis by Webb and Sheeran [27] concluded that a change in intention generally leads to a change in behaviour, albeit the effect size of the change in behaviour is often smaller than that of the change in intention. Recently, the TDF was revised by Cane and colleagues [28] which highlighted intention as a discrete domain impacting behaviour.

Using a validated scale developed based on a theoretical domain framework, this study explored the prevalence and relative impact of a comprehensive set of barriers, as well as intention, on hypothetical physical and mental health care access among CAF Health Services personnel.

## Methods

### Study population

Between May and July 2019, all CAF Health Services personnel (*N* = 3,448) were invited, via their work email, to complete a survey assessing barriers to care. Of the 3,448 eligible participants, 277 had invalid emails. Participants were excluded from the sample if they completed less than 50% of the items or if they were not employed in a health services role.

### Data collection

Participants were invited to complete an online questionnaire that included items measuring barriers, personal characteristics, and access to care using vignettes. After consenting to the study, participants were randomly assigned to complete one of two versions of the survey: access to care for mental health issues or access to care for physical health issues [24, 29]. Prior to completing the barrier items, participants read a prompt that indicated that the barrier items either related to seeking care for physical health issues exclusively or mental health issues exclusively. Aside from this, the 52-barrier items (see Additional file 1) were identical across the two surveys. Participants rated the extent to which the barrier item would prevent them from seeking care using a 6-point scale (1- Extremely Unlikely to 6- Extremely Likely).

All participants completed demographic, military characteristic, and health-related items across both versions of the survey. Intent to access care and self-rated health were survey specific (see below). Intent to access care was assessed through a single item: “When faced with a [physical/mental] health issue, I intend to access care” rated on a 7-point scale (1- Strongly Disagree to 7- Strongly Agree). Self-rated health was assessed using a single item: “In general, how would you rate your [physical/mental] health?”, rated on a 5-point scale (1- Very Poor to 5- Excellent).

### Primary outcome

In each version of the survey, participants were presented with two hypothetical four-step scenarios detailing symptoms related to: depression and post-traumatic stress disorder (PTSD) in the mental health survey; and pneumonia and back injury in the physical health survey (see Additional file 2). Each vignette was developed and vetted by the appropriate health professionals to ensure accurate progression of symptoms to the point where the individual should objectively seek formal care. At each step, participants were asked to choose which action they would take in the presented scenario. The responses were dichotomized at step three, the first step where access to care was the only appropriate action: not accessing care (i.e., choosing “I would do nothing/wait and see”, “I would self-treat”, or “I would informally consult a colleague or peer”) was coded as one, while accessing care (i.e., choosing “I would seek formal treatment using CAF health services”, or “I would seek formal treatment using civilian health services”), was coded as zero.

### Analysis

The prevalence of each barrier type was calculated as the percentage of participants who positively endorsed at least one item within a factor. The choice was made to dichotomize barrier score due to a non-linear relationship between the factors and care-seeking. Barrier items were grouped into eight barrier factors as defined in previous research by Frank and Born [24]. Table 1 provides a brief description of the factors and their psychometric properties. Barrier factors included: CAF HCP identity (e.g., If I accessed care, members of my unit might have less confidence in me as a health care provider), Discomfort accessing care at work (e.g., I’m uncomfortable receiving care from colleagues), Conflict with career goals (e.g., Accessing care would harm my career), Staffing and workload resources (e.g., My workload is too heavy for me to leave and access care), Knowledge and ability to access care (e.g., I don’t know how to access the services available to me), Organizational and social support (e.g., My chain of command discourages the use of health services), Treatment preferences (e.g., I want to solve the problem on my own rather than access care), and Concerns about confidentiality (e.g., When I seek care, my medical file may be seen by those who shouldn’t access it). Six barrier items that did not load onto factors were grouped into two additional categories: (1) Past experiences and expectations, and (2) Location related issues. These two additional factors were treated as individual characteristics in the analysis.

**Table 1:** Description of barriers to care

		Physical Health Sample (*n* = 530)		Mental Health Sample (*n* = 503)	
	# items	Factor Alpha	% Missing	Factor Alpha	% Missing
Barrier Factors					
CAF HCP identity	11	0.96	0.2	0.97	0.6
Discomfort accessing care at work	7	0.94	1.1	0.95	0.8
Conflict with career goals	6	0.91	0.8	0.92	0.8
Staffing and workload resources	5	0.89	0.2	0.90	0.4
Knowledge and ability to access care	4	0.90	1.9	0.88	2.6
Organizational and social support	4	0.91	3.2	0.86	3.0
Treatment preferences	5	0.85	0.4	0.86	1.6
Concerns about confidentiality	4	0.90	0.4	0.89	1.0
Additional Barrier Categories					
Past experiences and expectations	3	0.76	0.6	0.74	2.0
Location related issues	3	0.71	1.3	0.74	2.4

Barrier factors are listed in a column indicating the number of items per factor, with separate columns reporting the factor alpha and percent missing for the mental health and physical health samples

Intentions to access care was analyzed as a dichotomous variable with weak intentions (those reporting 1- Strongly Disagree to 5- Somewhat Agree) to the question “When faced with a [physical/mental] issue, I intend to access care” coded as one and strong intentions (those reporting 6- Agree and 7- Strongly agree) coded as zero. Self-rated health was also dichotomized to quantify the proportion in poor health, with poor health coded as one (those reporting 1- Very Poor or 2- Poor) and good health (those reporting 3- Fair to 5- Excellent) coded as zero.

Initially, we used bivariate analysis to calculate the crude odds ratio (crude OR) for not accessing care in each scenario separately for each type of barrier and for all individual characteristics. Then, odds ratios (aORs) were adjusted by logistic regression for variables considered a priori as potential confounders. To account for missing data, the “mi impute mvn” command in Stata [30] with an iterative Markov Chain Monte Carlo (MCMC) method [31] was used to augment data (30 imputations). The estimates, standard errors and 95% confidence intervals, were calculated using the “mi estimate” command [32]. Two models were computed for each outcome, one with and one without, intention to access care. Models were assessed using the largest fraction of missing information (FMI; for number of imputations ≥ 100 × FMI), average relative increases in variance (RVI; closer to zero the less effect missing data had on the variance estimate), and F-Tests (*p* < 0.05). All analyses were performed using Stata software (Version 14 [33];).

## Results

Of the 3,171 CAF personnel who received an invitation to complete the survey, 1270 individuals responded; however, 221 respondents were excluded as they had completed less than 50% of the items, and an additional 16 participants were excluded as they were not employed in a health services role. This yielded a final sample of 1033 participants (503 from the mental health survey and 530 from the physical health survey) with a response rate of 32.6%. See Table 2 for sample descriptions.

**Table 2:** Sample demographics

		Physical Health Sample (*n* = 530)		Mental Health Sample (*n* = 503)	
Variable	Categories	Count	%	Count	%
Gender	Female	259	48.9	236	46.9
	Males	242	45.7	234	46.5
Not reported/Missing		29	5.5	33	6.6
Age Group	20's	90	17.0	82	16.3
	30's	196	37.0	186	37.0
	40's	163	30.8	131	26.0
	50's +	59	11.1	77	15.3
	Missing	22	4.2	27	5.4
Preferred Language for Care	English/No reference	436	82.3	412	81.9
	French	75	14.2	64	12.7
	Missing	19	3.6	27	5.4
Rurality	Urban	291	54.9	228	45.3
	Peri-urban	70	13.2	65	12.9
	Rural	118	22.3	114	22.7
	Missing	51	9.6	96	19.1
Rank	JNCM	148	27.9	149	29.6
	SNCM	88	16.6	81	16.1
	Jr.Officer	175	33.0	146	29.0
	Sr. Officer	96	18.1	93	18.5
	Missing	23	4.3	34	6.8
Years of service	< 11 years	176	33.2	162	32.2
	11 to 20 years	204	38.5	201	40.0
	> 20 years	127	24.0	111	22.1
	Missing	23	4.3	29	5.8
Trade	Clinical core	300	56.6	255	50.7
	Clinical support	100	18.9	91	18.1
	Specialty	41	7.7	55	10.9
	Dental	57	10.8	49	9.7
	Missing	32	6.0	53	10.5
Poor self-rated health	Good	502	94.7	431	85.7
	Poor	28	5.3	72	14.3
	Missing	0	-	0	-
Access health care in the past	No	89	16.8	125	24.9
	Yes	439	82.8	373	74.2
	Missing	2	0.4	5	1.0
General intention to access care	Weak	303	57.2	296	41.2
	Strong	227	42.8	207	58.9
	Missing	0	-	0	-

*JNCM* Junior non-commissioned member, *SNCM* Senior non-commissioned member

Demographic variables are listed with separate columns reporting the count and percent for item categories the mental health and physical health samples

### Prevalence of barriers (Fig. 1)

**Fig. 1:**
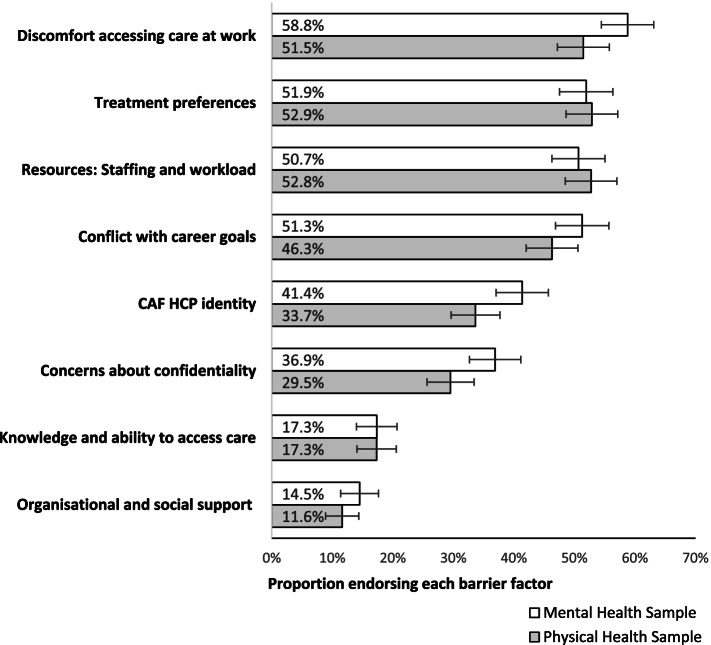
Proportion of respondents who endorsed barriers. Barrier factors are listed on the y-axis and the relative proportion of respondents are plotted as horizontal bars (with 95% confidence intervals) on the x-axis, with separate estimates plotted for in the mental health and the physical health samples

Each barrier factor had a similar prevalence in both the mental health and physical health samples. The most endorsed barrier factors in both surveys were: Discomfort accessing care at work, Treatment preferences (e.g., self-treat), Staffing and workload resources, and Conflict with career goals.

### Association between individual characteristics and hypothetical care-seeking (Table 3)

**Table 3:** Crude odds ratios of not accessing care in the scenarios by individual characteristics

Individual Characteristics			Pneumonia			Back Injury			Depression				PTSD	
			%	OR	*SE*	%	OR	*SE*	%	OR	*SE*	%	OR	*SE*
	Overall		25.3			18.0			46.7			28.7		
Gender	Females		29.5	R		20.1	R		45.3	R		28.5	R	
	Male		21.5	0.66*	0.14	15.8	0.75	0.18	48.3	1.13	0.21	29.2	1.03	0.21
Age Group	20's		30.0	R		16.9	R		47.6	R		30.5	R	
	30's		26.2	0.83	0.23	16.8	1.00	0.34	49.5	1.08	0.29	30.7	1.01	0.29
	40's		23.9	0.73	0.22	22.1	1.40	0.48	48.9	1.05	0.30	28.5	0.91	0.28
	50's +		20.3	0.60	0.24	15.3	0.89	0.41	37.7	0.67	0.22	22.4	0.66	0.24
Preferred Language for Care	English		26.6	R		19.7	R		49.8	R		29.0	R	
	French		18.9	0.64	0.20	9.5	0.43*	0.18	29.7	0.43**	0.12	26.6	0.88	0.27
Rurality	Urban		25.9			14.1	R		43.9	R		28.3	R	
	Peri-urban		25.7	0.99	0.30	15.7	1.13	0.42	47.7	1.17	0.33	32.3	1.21	0.37
	Rural		22.9	0.85	0.22	23.7	1.89*	0.52	53.5	1.47	0.34	28.1	0.99	0.25
Location related issues	No		21.7	R		15.3	R		41.8	R		24.1	R	
	Yes		39.8	2.38***	0.56	29.8	2.35**	0.60	67.7	2.92***	0.70	47.4	2.83***	0.67
Rank	JNCM		19.6			19.7			45.0	R		27.0	R	
	SNCM		26.1	1.45	0.46	17.1	0.84	0.29	53.1	1.38	0.38	27.5	1.02	0.32
	Jr. Officer		30.5	1.80*	0.48	17.7	0.88	0.25	46.6	1.07	0.25	30.1	1.16	0.30
	Sr. Officer		22.9	1.22	0.39	17.7	0.88	0.30	45.2	1.01	0.27	29.0	1.10	0.32
Years of service	< 11yrs		25.7			16.0			42.6	R		23.5	R	
	11 to 20 yrs		25.5	0.99	0.23	17.7	1.13	0.31	50.8	1.39	0.30	30.7	1.44	0.35
	> 20yrs		23.6	0.89	0.24	21.3	1.42	0.42	45.1	1.10	0.27	31.5	1.50	0.41
Trade	Core		24.8			20.0			43.9	R		29.5	R	
	Support		29.0	1.24	0.32	17.2	0.83	0.25	48.4	1.20	0.29	29.7	1.01	0.27
	Specialty		17.1	0.63	0.27	9.8	0.43	0.24	60.0	1.92*	0.58	31.5	1.10	0.35
	Dental		26.3	1.09	0.36	17.5	0.85	0.32	40.8	0.88	0.28	18.4	0.54	0.21
Past diagnosis	No		24.8	R		18.1	R		45.2	R		27.3	R	
	Yes		26.4	1.09	0.29	17.6	0.97	0.29	53.3	1.38	0.32	36.3	1.51	0.37
Poor Self-rated health	No		25.6	R		17.2	R		42.5	R		24.7	R	
	Yes		21.4	0.79	0.37	32.1	2.29	0.96	72.2	3.52***	0.99	52.8	3.41***	0.89
Access health care in the past	No		38.2	R		28.1	R		49.6	R		28.2	R	
	Yes		22.8	0.48**	0.12	16.0	0.49**	0.13	45.6	0.85	0.18	28.8	1.03	0.24
General intention to access care	Strong		13.9	R		8.3	R		25.6	R		13.5	R	
	Weak		40.7	4.31***	0.92	31.0	5.06***	1.28	61.5	4.63***	0.92	39.5	4.24***	1.00
Past experiences and expectations	No		19.2	R		12.4	R		37.3	R		20.1	R	
	Yes		42.1	3.06***	0.65	32.2	3.36***	0.76	69.8	3.88***	0.83	50.7	4.10***	0.89

% in category who did not access care in given scenario, *R* reference category, *SE* standard error, *JNCM* Junior non-commissioned member, *SNCM* Senior non-commissioned member

^***^p < 0.001, **p < 0. 01, *p < 0.05

Individual characteristics are listed with separate columns reporting the percent who did not access care in each variable category, the crude odds ratio (OR) of not accessing care with respect to the reference category (R), and the standard error (SE) of the odds ratio for each of the four vignettes depicting symptoms of pneumonia, back injury, depression, and PTSD

Compared to males, females had significantly higher crude odds of not accessing care for pneumonia-like symptoms in the scenarios. Those who prefer to access care in English had higher crude odds of not accessing care for the back injury scenario and for the depression scenario compared to those who prefer to access care in French. Several associations were noted across military characteristics: the crude ORs were higher for those posted to rural locations, compared to those posted to urban areas for the back injury scenario; higher for junior officers, compared to junior non-commissioned members for the pneumonia scenario; and higher for those in specialty occupations, compared to core clinical trades for the depression scenario. Those with poor self-rated mental health had higher crude ORs in both mental health scenarios compared to those reporting better self-rated health. The crude ORs in the physical health scenarios did not differ significantly by self-rated physical health. Those reporting weak general intentions to access care had higher crude ORs across all scenarios. The two additional factors, associated with location related service issues, and experience and expectations about care, also produced significant estimates across all scenarios.

### Crude association between barriers and hypothetical care-seeking (Fig. 2)

**Fig. 2:**
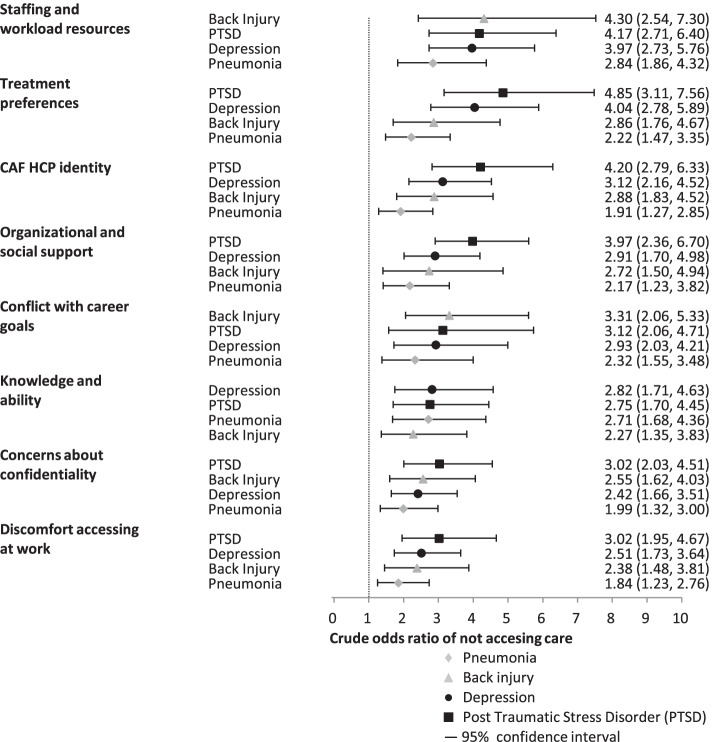
Crude odds ratio of not accessing care in the vignettes by barrier factor. Barrier factors are listed on the y-axis while the crude odds ratio estimates for not accessing care (with 95% confidence intervals) are plotted on the x-axis, for each of the four vignettes depicting symptoms of pneumonia, back injury, depression, and PTSD. Estimates are sorted from largest to smallest within each factor and overall

All eight barrier factors were significantly associated with not seeking care in all four vignettes. Specifically, endorsement of a barrier factor was associated with statistically higher crude odds of not accessing care in all the scenarios, compared to those who did not endorse the barrier factor. Staffing and workload resources and Treatment preferences (e.g., self-treat) were the strongest predictors of not accessing care in the mental health scenarios. Staffing and workload resources and Conflict with career goals were the strongest predictors of not accessing care in the physical health scenarios. Discomfort accessing care at work, Concerns about confidentiality, and Knowledge and ability to access care were the weakest barriers associated with not accessing care across all the scenarios. Crude OR estimates ranged from 1.87 to 2.91 for the pneumonia vignette, 2.24 to 4.53 for the back injury vignette, 2.48 to 4.03 for the depression vignette, and 2.74 to 4.71 for the PTSD vignette.

### Adjusted odds of hypothetical care-seeking (Tables 4 and 5)

**Table 4:** Imputed logistic regression modelling: Physical health scenarios with and without intentions

Model Variables	Pneumonia		(with intentions)		Back Injury		(with intentions)	
	aOR	*SE*	aOR	*SE*	aOR	*SE*	aOR	*SE*
Individual characteristics								
Female gender	1.58†	0.37	1.49	0.36	1.44	0.38	1.40	0.38
Age	0.73*	0.10	0.73*	0.11	1.15	0.18	1.19	0.19
Prefer accessing care in English	1.22	0.420	1.03	0.37	1.29	0.55	1.09	0.48
JNCM	0.42**	0.12	0.37**	0.11	0.90	0.28	0.83	0.27
Core trade	0.93	0.22	0.84	0.21	1.11	0.30	1.07	0.30
Stationed in rural location	0.74	0.21	0.71	0.21	1.55	0.47	1.55	0.49
Location related issues	1.41	0.39	1.30	0.37	1.22	0.36	1.14	0.35
Poor self-reported health	0.63	0.33	0.65	0.35	1.57	0.74	1.70	0.84
Accessed care in the past	0.41*	0.11	0.50*	0.14	0.40**	0.12	0.47*	0.15
Past experiences and expectations	2.28*	0.6	2.01*	0.63	1.65	0.53	1.44	0.47
Weak general intentions			3.39***	0.82			3.28***	0.91
Barrier Factors								
CAF HCP identity	0.72	0.23	0.66	0.22	1.07	0.36	1.03	0.35
Discomfort accessing care at work	0.76	0.23	0.79	0.25	0.79	0.28	0.80	0.29
Conflict with career goals	1.58	0.43	1.44	0.40	1.82†	0.57	1.67	0.53
Staffing and workload resources	1.93*	0.55	1.62	0.48	2.29*	0.67	1.93†	0.67
Knowledge and abilities	1.23	0.38	1.28	0.41	0.91	0.31	0.93	0.32
Organizational and social support	1.17	0.44	1.22	0.48	0.90	0.34	0.95	0.38
Treatment preferences	1.38	0.37	1.24	0.34	1.48	0.46	1.31	0.41
Concerns about confidentiality	0.95	0.29	1.05	0.33	1.02	0.33	1.10	0.37
Model constant	0.77	0.51	0.57	0.39	0.05***	0.04	0.03***	0.02
Model Descriptors								
n	530		530		503		503	
Average RVI^a^	0.0238		0.0278		0.0266		0.0320	
Largest FMI^b^	0.0961		0.1044		0.1684		0.1696	
Model F Test	F( 18.869979.5) = 3.45		F(19,674,789.5) = 3.34		F(18,690,448.7) = 2.84		F(19,515,145.5) = 4.45	
*p*	< 0.0001		< 0.0001		< 0.0001		< 0.0001	

^a^M ≥ 100 × FMI, in all models where M (number of imputations) is sufficient

^b^ The closer the RVI is to zero, the less effect missing data have on the variance estimate, in this case all RVI are low, *JNCM*- Junior non-commissioned member; ***p < 0.001, **p < 0.01, *p < 0.05, † *p* ≤ 0.06

Model variables are listed in a column and the adjusted odds ratio (aOR) estimate for not accessing care with standard error (SE) are reported in separate columns for the two physical health vignettes: pneumonia and back injury

**Table 5:** Imputed logistic regression modelling: Mental health scenarios with and without intentions

Model Variables	Depression		(with intentions)		PTSD		(with intentions)	
	aOR	*SE*	aOR	*SE*	aOR	*SE*	aOR	*SE*
Individual characteristics								
Female gender	0.75	0.17	0.77	0.18	0.78	0.19	0.80	0.20
Age	0.87	0.11	0.93	0.12	0.84	0.12	0.87	0.12
Prefer accessing care in English	2.49**	0.85	1.82	0.66	1.16	0.41	0.86	0.32
JNCM	0.75	0.20	0.76	0.21	0.75	0.22	0.75	0.22
Core trade	0.63†	0.15	0.62†	0.15	0.94	0.23	0.95	0.24
Stationed in rural location	1.33	0.36	1.52	0.42	0.77	0.22	0.86	0.25
Location related issues	1.36	0.40	1.39	0.43	1.12	0.33	1.10	0.33
Poor self-reported health	2.27*	0.77	2.14*	0.75	1.55	0.49	1.47	0.48
Accessed care in the past	0.66	0.16	0.74	0.19	0.75	0.20	0.83	0.23
Past experiences and expectations	1.61	0.46	1.60	0.48	1.52	0.44	1.52	0.45
Weak general intentions			3.38***	0.78			2.84***	0.75
Barrier Factors								
CAF HCP identity	0.91	0.27	0.84	0.26	1.41	0.45	1.34	0.43
Discomfort accessing care at work	0.93	0.25	0.77	0.22	0.98	0.30	0.85	0.26
Conflict with career goals	1.59†	0.42	1.65†	0.45	1.20	0.35	1.23	0.36
Staffing and workload resources	2.52***	0.60	2.64***	0.65	1.98**	0.52	2.01**	0.54
Knowledge and abilities	1.01	0.31	0.92	0.30	1.05	0.31	0.96	0.29
Organizational and social support	0.93	0.32	0.88	0.32	1.45	0.47	1.43	0.48
Treatment preferences	1.97*	0.47	1.79*	0.44	2.35**	0.63	2.17**	0.59
Concerns about confidentiality	1.19	0.35	1.15	0.35	1.15	0.35	1.13	0.35
Model constant	0.34	0.12	0.18*	0.12	0.24**	0.16	0.14**	0.10
Model Descriptors								
n	503		503		503		503	
Average RVI^a^	0.0420		0.0394		0.0347		0.0320	
Largest FMI^b^	0.2159		0.1933		0.1813		0.1696	
Model F Test	F(18,288,686.3) = 4.92		F(19,344,713.2) = 5.41		F(18,416,726.1) = 4.29		F(19,515,145.5) = 4.45	
*p*	< 0.0001		< 0.0001		< 0.0001		< 0.0001	

^a^*M* ≥ 100 × FMI, in all models where *M* (number of imputations) is sufficient

^b^ The closer the RVI is to zero, the less effect missing data have on the variance estimate, in this case all RVI are low; JNCM- Junior non-commissioned member; ***p < 0.001, **p < 0.01, *p < 0.05, † *p* ≤ 0.06

Model variables are listed in a column and the adjusted odds ratio (aOR) estimates for not accessing care with standard error (SE) are reported in separate columns for the two mental health vignettes: depression and PTSD

In the model that included intentions, holding all other variables constant, the odds of not accessing mental health care were significantly higher for those endorsing barriers related to resources (aOR_depression_ = 2.64; aOR_PTSD_ = 2.01) and treatment preferences (e.g., self-treat) (aOR_depression_ = 1.79; aOR_PTSD_ = 2.17). None of the eight barrier factors were significant predictors of accessing physical health care (at *p* < 0.05). Weak general intentions to access care was the only statistically significant predictor in the model across all scenarios (aOR_pneumonia_ = 3.39; aOR_back injury_ = 3.28; aOR_depression_ = 3.38; aOR_PTSD_ = 2.84). Age and rank were the only demographic and military characteristic predictors with statistically significant aORs, but these differences were only noted for not accessing care in the pneumonia scenario. Having accessed care in the past was significantly associated with not accessing care in the physical health scenarios (aOR_pneumonia_ = 0.50; aOR_back injury_ = 0.47), but not in the mental health scenarios, where past care predicted increased likelihood of accessing care. Poor health predicted not accessing care in the depression scenario (aOR_depression_ = 2.14), while the past experiences and expectations factor predicted not accessing care only in the pneumonia scenario (aOR_pneumonia_ = 2.01). Models not controlling for weak intentions to access care presented similar results.

## Discussion

The goal of this paper was to examine the prevalence and relative impact of a wide array of barriers on hypothetical care-seeking for both mental and physical health care.^1^ Examination of the endorsement of barrier factors revealed that discomfort accessing care at work, treatment preferences (e.g., self-treat), staffing and workload resources, and conflict with career goals were all endorsed by approximately half of respondents across both the mental health and physical health surveys. Although there were some differences between the degree of endorsement of the factors between the surveys versions, the estimates generally overlapped. This finding reproduced the conclusions of a recent study by Britt et al., [16], that, although there are some differences between the reported barriers to accessing mental and physical health care, there are more similarities.

### Independent links between barrier factors and hypothetical care seeking

All the barrier factors significantly increased the odds of not accessing care in both the mental and physical health scenarios. Of note, the crude odds ratios of not seeking care for barriers related to self-treatment, identity, and resources were reasonably large, with some crude ORs above four, indicating that the odds of not accessing formal care when needed in the scenarios was at least four times higher for those who reported these barriers compared to those who did not. This finding is both statistically and clinically significant, and these estimates are divergent from those for actual care-seeking propensity published in other studies of military populations (e.g., Sudom et al., noted crude ORs between 0.7 and 1.6 for all measured barriers [17] noting that negative attitudes were negatively associated, while stigma and structural barriers were positively associated, with hypothetical access to care). Also of note, aside from Staffing and workload resources and Treatment preferences (e.g., preference to self-treat), the barrier factors with the highest endorsement were not the most impactful. Together these findings suggest that perceived impact of barriers may not be as predictive of behaviour as previously thought. For example, barriers related to the discomfort accessing care at work were highly endorsed but were not the strongest predictors of avoiding accessing care in the scenarios. Our results suggest that although respondents perceived accessing care at their workplace as a key barrier, many respondents who endorsed this barrier still indicated they would access care in the scenarios.

### Association controlled for the demographic, military, and health variables

Then we modelled the association between the barrier factors and the health scenarios while controlling for the demographic, military, and health variables. Weak intent to access care was consistently the strongest predictor of not accessing both physical and mental health care in the scenarios, even when controlling for other barriers, demographic, military, and health variables. The findings appear to be in line with many studies that have reported that intention is a consistent predictor of health care providers’ professional behaviour [34, 35].

The literature suggests that cognitive factors (such as beliefs about capabilities, beliefs about consequences, social influences, professional role, and identity), past behaviour, emotion, and to a lesser extent, environmental influences, and knowledge often influence a health care professional’s intentions towards a variety of behaviours such as care seeking [34]. In other words, the other barrier factors included in this study may be indirectly impacting care seeking through their association with intent to access care. Thus, interventions targeting barriers related to these domains may concurrently reduce barriers and improve intentions to access care which should subsequently improve access to care.

Interestingly, none of the barrier factors significantly increased the odds of hypothetically seeking physical health care in the adjusted models. However, staffing and workload resources was a marginally significant predictor of the likelihood of not accessing care for the back pain scenario, but not for the pneumonia scenario. It is important to note that though we have created distinct barrier factors, they are derived from theoretical domains that have conceptual overlap. As an example, barriers in the organizational and social support factor relate to how the perceptions or actions of others influence whether an individual accesses care, which could also impact one’s identity (CAF HCP identity factor). Though there were no issues with multicollinearity in the model (and no univariate correlations greater than 0.60), the conceptual overlap between the factors may be contributing to the lack of significant findings in the model. As discussed above, barriers may be indirectly influencing access to care in the scenarios through intent to access care. Even if the barrier factors were not significant in the adjusted model, they did have strong associations with hypothetical physical care seeking when examined alone. Additionally, the adjusted models themselves were significant, suggesting that the barriers are playing a role.

Endorsing Staffing and workload resources significantly increased the odds of not accessing care across both mental health conditions. This barrier factor includes barriers such as not having time to access care and not wanting to leave their colleagues short staffed. Treatment preferences (e.g., preference to self-treat) also was a significant predictor in the adjusted mental health models (but not in the physical health models). This barrier factor includes barriers such preferring to solve problems on their own, preferring to treat others first, or anticipating that a problem will get better on its own. This finding is in line with research that has found higher estimates for the preference to self-treat in the mental health context compared to the physical health context in samples of military members [16] and healthcare providers [36].

As mentioned above, the lack of significance in the adjusted model does not necessarily indicate that no other barriers are impactful. However, the findings do suggest that resources and preferences to self-treat are strong barriers when considering whether to access mental health care. Studies have used behavioural change techniques such as mental rehearsal and goal setting/planning to address motivation constructs [19] and evidence suggests that providing information about the consequences of not accessing care could target attitudinal and emotional barriers related to the preference to self-treat [19, 37]. However, we must remember that accessing care is not a single action. Behaviour often involves a complex sequence of actions over time [38] and the barriers may differ at each step. For example, a CAF health care provider may need to first recognise the need for formal care, deal with resource issues when scheduling a time to seek care, then face privacy concerns to attend care at their place of work. The impact of a barrier on a behaviour may be attenuated by one or more barriers acting at a different step in the access to care pathway [38]. Just as the barriers encountered at each step differ, so might the processes required to change behaviour. Interventions that target barriers risk not improving access to care behaviour if other barriers in the sequence are not also addressed or poorly defined. Recommendations by Presseau and colleagues highlight that the action, actor, context, target, and time should be detailed for each behaviour [39]. Therefore future studies should explore and define the steps to accessing to care in order to determine the sequencing of step-specific barriers in the access to care pathway.

### Stigma

Though much of the literature has focused on stigma as the top barrier to mental health care in military populations [25], more recent studies have noted the relative importance of barriers related to intentions [16], attitude towards care [17], and capabilities (such as resources, support and structural barriers [17]). Stigma related items were classified under the CAF HCP identity factor, (including items such as “I feel that others will discriminate against me if I access care” and “I feel embarrassed when I have to access care”). The unadjusted odds ratio for CAF HCP identity predicting not accessing care for mental health was relatively high. However, CAF HCP identity was not one of the most endorsed factors and, in the adjusted models, was not a significant predictor of hypothetical access to care in any of the scenarios. This suggests that though stigma remains an important construct to consider, its influence on hypothetical behaviour may be indirect or simply an indicator of motivation.

### Limitations

Several sources of bias may have resulted from the study methods and design. Though the use of multiple imputation to account for missing data is a strength of this study, it is also has certain limitations. Multiple imputation allows for limited types of analyses and, in the case of logistic regressions, there are limited model fit statistics available. However, we believe the strength of imputing missing data is stronger than the limitation of restricted analytical options and outputs.

Although we were able to impute missing data, we must note that less than half of the target population completed the survey. Therefore the results may not be generalizable to the entire CAF health care provider population. We expect that the experience of certain types of barriers may also be associated with non-participation. For example, those who experience resource issues, such as lack of time to seek care, likely also lack the time to complete a survey. Another limitation is that some of the barriers are specific to CAF HCPs (e.g., healthcare provider identity or accessing care at work), and may differ from those experienced by other military trades or civilian providers, particularly those who do not seek treatment where they are employed. However, the items could be modified (e.g., items removed or reworded) to better reflect barriers experienced by other populations. Furthermore, we designed this study using the original TDF [22], with 12-domains. We measured intention using a single item similar to what had been used in other research (e.g., Squires and colleagues [40]). However, the revised TDF by Cane and colleagues [28] includes intention as a discrete domain. Thus, intention should have been assessed using multiple items as recommended by as Huijg and colleagues [41].

As with most research, all predictors and outcomes were self-report and thus subject to known biases [42, 43]. We were not able to measure actual care seeking behaviour. The literature suggests that subjective measures (i.e., self-report) are more effective at predicting a self-reported behaviour outcome than objective measures [44], thus participant’s predicted behaviour in the hypothetical scenarios is likely not a perfect reflection of what they would actually do in that scenario [43–45] and may inflate the proportion of variance explained in the models. Future studies should explore whether the results of this study are reproducible using measures of actual behaviour.

### Implications

Despite the limitations, this is the first published example of a study that has quantified the impact of a variety of barriers to care developed using the TDF. Interventions designed to change behavior rely on, and are heavily impacted by, the accuracy of assessments. Therefore, employing effective methodologies [46, 47] to identify barriers and can lead to better interventions. Unique to this study, we were able to demonstrate the relative impact of a breadth of theory-based barrier factors on hypothetical care-seeking across a variety of health contexts (i.e., two discrete mental health issues; and both an acute and chronic physical health issue). The use of vignettes helps to define the context of the behaviour [33, 48], allows us to highlight key differences in actions between health conditions. Studies that are too narrowly focused on a single type of barrier, or context overlook important factors and miss opportunities to intervene. For example, preference to self-treat significantly impacted hypothetical care-seeking for mental health issues. Other research on barriers to care, has not included these types of barriers before. Thus, any recommended interventions would fail to address this barrier type.

Additionally, the use of theory-based barrier factors provides a deeper understanding of access to care issues using established methods and psychological constructs [22, 23, 28]. This research will enable the development of context specific and evidence-based interventions to improve access to care by targeting psychosocial constructs [19] that, unlike demographics, are amenable to change. For example, our results suggest that interventions should target motivation (such as one’s intention to self-treat and conflicts with career goal) and opportunity (such as context specific induced lack of time) in order to improve access to both physical and mental health care. The behavioural change literature proposes goal setting could address barriers related to motivation [19]. Specifically, having health care providers set health-related goals may increase their intention to access care. Modelling behaviours is another intervention that targets motivational barriers. Specifically, ensuring that senior HCPs access care when needed and model care seeking behaviour should increase intention to access care. Additionally, educating health care providers on the consequences of self-treatment may address barriers related treatment-preference [19]. Staffing and workload resources are likely better addressed at the organizational-level. Specifically, providing health care personnel with mandated time off to access care may alleviate opportunity barriers.

## Conclusion

Our findings suggest that intention to access care is the strongest predictor of care seeking in hypothetical situations. A lack of resources and preference to self-treat directly and negatively influence access to care in the vignettes. Additionally, other barriers may potentially be influencing access to care in the scenarios, indirectly through intent to access care or at different points along the access to care pathway. Future studies should qualitatively explore the sequence of behaviours needed to access care and quantify the indirect associations between barriers and behaviour, exploring the mediating effects of intentions. Given the findings, it appears that the best strategy moving forward is to target both intent to access care as well as barriers that may be impacting intent to access care (i.e., preference to self-treat and staffing and workload). Using evidence based behaviour change techniques to target these barriers, such as education, modelling behaviour, goal setting, and offering protected time to access care should increase access to care. Using a multi-prong theoretical approach to barrier reduction provides the best opportunity for behavioural change and increasing access to care.

## Supplementary Information

**Additional file 1. **Barrier items from the survey.

**Additional file 2. **Survey scenarios.

## Data Availability

The data sets used and/or analysed during the current study are subject to the Access to Information Act and can be requested from the Government of Canada, through an access to information request online https://atip-aiprp.tbs-sct.gc.ca/ or by contacting the National Defence Access to Information and Privacy Coordinator at ATIP-AIPRP@forces.gc.ca.

## Abbreviations

AOR: Adjusted odds ratio
CAF: Canadian Armed Forces
COM-B system: Capability, Opportunity, Motivation, Behaviour
crude OR: Unadjusted odds ratio
FMI: Fraction of missing information
HCPs: Health care providers
JNCM: Junior non-commission member
PTSD: Post-traumatic stress disorder
RVI: Relative increases in variance
SNCM: Senior non-commission member
SE: Standard error of the mean
TDF: Theoretical Domains Framework

## Footnotes

1 In the following discussion we will use the phrases “care seeking” or “access to care,” but wish to clarify that the use of those phrases refer to hypothetical behaviour, and not actual behaviour based on our measurements.
