# Supplementary material for: The relative impact of barriers to care among military health services personnel: exploring differences using context specific scenarios
Source: BMC Health Serv Res. 2022 May 6;22:607. doi: 10.1186/s12913-022-07850-5 (PMC9074225; doi:10.1186/s12913-022-07850-5)
Supplement: Supplementary file 1 — Additional file 1. Barrier items from the survey. [file 12913_2022_7850_MOESM1_ESM.docx]

Additional file 1: Barrier items from the survey

Response Options:

To what extent do you agree with this statement?

| Strongly Disagree | Disagree | Somewhat Disagree | Somewhat Agree | Agree | Strongly Agree | I don’t know | Not Applicable |
| --- | --- | --- | --- | --- | --- | --- | --- |

To what extent would this prevent you from seeking care?

| Extremely Unlikely | Very Unlikely | Somewhat Unlikely | Somewhat Likely | Very  Likely | Extremely Likely | I don’t know | Not Applicable |
| --- | --- | --- | --- | --- | --- | --- | --- |

1) Accessing care would harm my career.

2) If I were being deployed, accessing care would prevent me from deploying.

3) Accessing care will prevent me from receiving a posting that I want in the future.

4) Accessing care will harm my future chances of promotion.

5) If I were going on course, seeking care would prevent me from being able to go.

6) Accessing care can result in my being medically released.

7) I don’t have time to access care.

8) When I access care, I don’t have control over my care plan.

9) I’m not afforded that same privacy as other patients because my colleagues are part of my circle of care.

10) Because I access care where I work, when I seek care, people notice or find out I was there.

11) I have concerns about the confidentiality of the information I share.

12) If I have to access care, I’m concerned about being perceived as someone who is taking advantage of the system (“faking”).

13) If I access care, my subordinates, and/or my superiors may doubt my competency as a health care provider.

14) I feel embarrassed when I have to access care.

15) I feel that others treat me differently if I access care.

16) I feel that others will discriminate against me if I access care.

17) I don’t know what services are available to me.

18) I don’t know how to access the services available to me.

19) I’m unfamiliar with any policies that pertain to seeking care in the CAF (e.g., accessing care externally to CAF or switching CDUs).

20) I find it difficult to navigate the administrative processes necessary to seek some types of care (e.g., MH, civilian care).

21) My workload is too heavy for me to leave, and access care.

22) I don’t want to leave my colleagues short-staffed to go access care.

23) I would have difficulty getting time off to access care.

24) I would think less of myself as a health care provider if I couldn’t solve my own health problems.

25) My leadership abilities may be called into question if I accessed care.

26) Seeking care may undermine my authority in the eyes of my subordinates.

27) If I accessed care, members of my unit might have less confidence in me as a health care provider.

28) If I accessed care, my unit leadership might perceive me as less competent.

29) If I accessed care, I would be seen as weak.

30) I want to solve the problem on my own rather than access care.

31) When I am sick, I think the problem might get better by itself.

32) I believe that professional care probably will not be more helpful than what I can do myself.

33) It’s my role as a health care professional to treat others first.

34) My immediate supervisor does not support my accessing health services.

35) My chain of command discourages the use of health services.

36) My Commanding Officer does not value my health.

37) My colleagues (of similar rank) would not support my decision to access health care if needed.

38) I find it challenging to be in the role of patient.

39) I believe my appointments are less formal (e.g., I’m spoken to more casually than other patients; appointments are squeezed in) than the appointments other patients experience.

40) The provincial health care system impacts the quality of care I receive from CAF health services (i.e., relating to services received on the civilian side).

41) I’m uncomfortable receiving care from colleagues.

42) I’m uncomfortable receiving care from my subordinates.

43) I’m uncomfortable receiving care from my superiors.

44) I’m uncomfortable accessing care where I work.

45) It makes me uncomfortable to sit in the waiting room with patients while waiting to be seen.

46) Where I am located, I have limited access to care.

47) Organizing transport to access care off base would be difficult.

48) If I need to access care, I am not replaceable.

49) I have had past negative experiences when accessing care.

50) When I access care, my colleagues are able to see why I’ve sought care in the past.

51) When I seek care, my CFHIS file may be seen by those who shouldn’t access it.

52) I don’t believe that there is sufficient monitoring for appropriate access of CFHIS files.
