# Supplementary material for: The relative impact of barriers to care among military health services personnel: exploring differences using context specific scenarios
Source: BMC Health Serv Res. 2022 May 6;22:607. doi: 10.1186/s12913-022-07850-5 (PMC9074225; doi:10.1186/s12913-022-07850-5)
Supplement: Supplementary file 2 — Additional file 2. Survey scenarios. [file 12913_2022_7850_MOESM2_ESM.docx]

Additional file 2: Survey scenarios

In this next section, you are asked to read the scenarios, and indicate which action you would be most likely to take given the information, and your current situation.

Response Options:

a. I would do nothing/I would wait, and see

b. I would self-treat

c. I would informally consult a colleague or peer

d. I would seek formal treatment using CAF health services

e. I would seek formal treatment using civilian health services (bypass CAF services)

**PH Scenario A: Pneumonia**

Part I:

You wake up in the morning, and notice that you have a sore throat, and a slight non-productive cough. The symptoms persist through the day, and have not resolved when you wake up the following day.

Part II:

By the second day, your symptoms continue, and now your cough has become productive, and you are developing a low grade fever. You’ve also noticed that your breathing is more laboured.

Part III:

Your cough has progressed (discoloured sputum), and worsened. You continue to experience a fever, and you are now also experiencing chills, and muscle pains. You also hear a crackling sound when you inhale/exhale.

Part IV:

You still have a fever, and now you’re also vomiting. You still have a productive cough with laboured breathing accompanied by a crackling sound.

**PH Scenario B: Back Injury**

Part I:

While helping to mobilize a patient, you experience some discomfort in your lower back. Later that day, the discomfort is still present with the pain increasing, and decreasing throughout the day.

Part II:

A week later, the pain is still present, and, though it does come, and go, it is generally persistent. You experience pain if you sit or stand in the same position for long periods of time. Your sleep has also been disrupted because of the pain.

Part III:

It’s now been two months since the injury, and you’re still experiencing pain. Some days it’s worse than others. You’re unable to sit or stand for long periods of time without pain.

Part IV:

Six months have passed, and the back pain comes, and goes. Some days it’s better than others. You find your ability for normal daily activity to be reduced, and you’re unable to perform your duties without discomfort.

**MH Scenario A: Depression**

Part I:

Work has been stressful lately, and you find yourself with less energy than usual. You feel like your work performance is really slipping lately, and you’re having trouble concentrating on your tasks. You don’t find that work is as enjoyable or interesting as you usually do. You’re still engaging in typical activities at home, but because of exhaustion, it takes more effort than it normally does.

Part II:

A week has passed, and work has settled down; however, you still find your energy affected. A few colleagues have noted that you’re finishing tasks more slowly than you normally do, and you feel guilty that you’re letting them down. You’re still having trouble concentrating at work, and feel indecisive when asked to weigh in. Work just isn’t enjoyable anymore, and when you go home, you feel tired, and a bit empty. You’ve started to do less around the house (e.g., doing fewer chores around the house) in favour of getting into bed earlier.

Part III:

It’s been two weeks, but you haven’t returned to normal. You’ve been taking sick days but, when you do go to work, you feel useless, and that you’re more of a burden than a help. You still don’t have any energy, and it isn’t helped by the fact you’re having trouble sleeping at night, and fighting the urge to sleep during the day. Throughout most of the day, you find yourself feeling sad or empty. At home, you’re mostly inactive, laying on the couch watching TV. You don’t have the drive or interest to do anything else.

Part IV:

It’s been about a month, and you think you’re getting worse. Your mood, for one, has worsened to the point where you feel depressed, and hopeless almost all of the time. You’re not finding pleasure in any of the things you did before. Work is no longer interesting. Your sleep is still disturbed. You’re still going to work, but your performance is poor by your own estimation, and it still takes you longer to complete tasks that you used to be able to do in half the time. Your focus is off, and you’re just moving slower. You’re not sure of the point of you being there at all. You’ve stopped going out because it’s too exhausting, and overwhelming, so you continue spending a lot of time alone.

**MH Scenario B: PTSD**

Part I:

A week ago, you returned from a disaster relief effort in a densely populated area that resulted in many casualties. It was a highly demanding deployment, and you’re still thinking about your experience while you were over there. You’ve noticed yourself becoming more tense than usual. There doesn’t seem to be a reason for your tension, and yet it continues to persist, making you a bit more irritable than normal.

Part II:

Another week passes, and you continue to feel tense, and irritable. You also feel hyperaware of everything around you which makes it hard for you to concentrate. When your tenseness does calm down, you find yourself feeling nothing, almost like a numbness. You’re less interested in work, and you find your ability to concentrate diminishing. Being around others is more grating than usual, you find yourself feeling angry, and annoyed at little things.

Part III:

It’s been over a month, and you find yourself thinking about your relief efforts, and picturing the devastation you saw. When people ask you about your deployment, you tell them that you’d rather not discuss it. In fact, you find yourself pushing away or detaching from friends, and family. With your alternating tenseness, and numbness continuing, you’re finding it hard to connect with other people anyways. Plus, you’re agitated a lot of the time, and you become irritated easily. You’ve started to experience insomnia, and, when you do sleep, you have nightmares.

Part IV:

It’s been about three to six months, you’re still irritable, and lately that has manifested in angry outbursts over trivial matters. You continue to feel detached from others including your closest family, and friends. In between feelings of anger, you feel nothing, and find yourself unable to enjoy your hobbies. You continue to have intrusive thoughts where you think about your disaster relief experience, and you find you’ve started having nightmares about it. You’ve also found that crowded spaces make you feel tense, and you now avoid going to places where there might be large crowds. You realize that, based on what you saw, you think that generally people can’t be trusted.
